# Searching for airways biomarkers useful to identify progressive pulmonary fibrosis

**DOI:** 10.1186/s12890-023-02714-y

**Published:** 2023-10-26

**Authors:** Piera Soccio, Giorgia Moriondo, Giulia Scioscia, Valentina Leo, Pasquale Tondo, Luciana Salerno, Paolo Palange, Maria Pia Foschino Barbaro, Donato Lacedonia

**Affiliations:** 1 Department of Medical and Surgical Sciences, University of Foggia, Viale Luigi Pinto, 1, Foggia, 71122 Italy; 2 Institute of Respiratory Diseases, Policlinico of Foggia, Viale Luigi Pinto, 1 , Foggia, 71122 Italy; 3 Department of Public Health and Infectious Diseases, Sapienza University of Rome, Viale Università, 37, Rome, 00185 Italy; 4 Division of Pulmonary Medicine, Policlinico Umberto I Hospital, Viale del Policlinico, 155, Rome, 00161 Italy

**Keywords:** IPF, Progressive pulmonary fibrosis, microRNA, Exosome, Exo-miRNA, Biomarkers

## Abstract

**Background:**

Idiopathic pulmonary fibrosis (IPF) is a chronic and progressive disorder with unknown etiology. To date, the identification of new diagnostic, prognostic and progression biomarkers of IPF turns out to be necessary.

MicroRNA (miRNA) are small non-coding RNAs which negatively regulate gene expression at the post-transcriptional level in several biological and pathological processes. An aberrant regulation of gene expression by miRNA is often associated with various diseases, including IPF. As result, miRNAs have emerged as potential biomarkers with relevance to pulmonary fibrosis.

Several reports suggested that miRNAs are secreted as microvesicles or exosome, and hance they are stable and can be readily detected in the circulation. In the contest of miRNAs as circulating biomarkers, different studies show their role in various types of interstitial lung diseases and suggest that these small molecules could be used as prognostic markers of the disease.

Exosomes are small, lipid-bound vesicles able to carry various elements of the naïve cells such as proteins, lipids, mRNAs and miRNA to facilitate cell communication under normal and diseases condition. Exosomal miRNAs (exo-miRNA) have been studied in relation to many diseases. However, there is little or no knowledge regarding exo-miRNA in bronchoalveolar lavage (BAL) in IPF.

Our study's aim is to evaluate the changes in the expression of two exo-miRNAs in BAL, respectively miR-21 and miR-92a, through highlighting the differences between IPF, progressive pulmonary fibrosis (PPF) and not-progressive pulmonary fibrosis (nPPF).

**Methods:**

Exosomes were characterized by Western Blot and Multiplex Surface Marker Analysis. Exosomal miRNA expression was performed by qRT-PCR. ANOVA or Kruskal–Wallis test, based on data normality, was used to compare the differential expression between groups.

**Results:**

MiR-21 expression was significantly higher in the nPPF group than in both IPF and PPF. A result that could point above a possible role of miR-21, as a biomarker in the differential diagnosis between PPF and nPPF. MiR-92a, indeed, was down regulated in PPF compared to IPF and down regulated in PPF compared to nPPF.

**Conclusions:**

This study demonstrated the putative role of both miR-21 and miR-92a as possible biomarkers of pulmonary fibrosis progression. Moreover, the role of exo-miRNAs is examined as a possible future direction that could lead to new therapeutic strategies for the treatment of progressive and non-progressive pulmonary fibrosis.

**Supplementary Information:**

The online version contains supplementary material available at 10.1186/s12890-023-02714-y.

## Background

Idiopathic pulmonary fibrosis (IPF) is the most common form of idiopathic interstitial pneumonia. It is a rare, chronic, progressive, fibrosing interstitial lung disease of unknown aetiology and cure, which is characterised by a radiological-histopathological pattern of UIP (Usual Interstitial Pneumonia) [1]. IPF is characterized by alveolar epithelial cell injury and activation, infiltration of inflammatory cells, initiation of epithelial mesenchymal transition (EMT), aberrant proliferation and activation of fibroblasts, exaggerated deposition of extracellular matrix (ECM) proteins, and finally leading to the destruction of lung parenchyma.

Similarity to IPF, other interstitial lung diseases can develop progressive pulmonary fibrosis (PPF) characterized by declining lung function, a poor response to immunomodulatory therapies and early mortality. PPF is defined as the presence of at least two of the following criteria: worsening respiratory symptoms, functional decline (decline in FVC > 5% predicted or absolute decline in DLCO ≥ 10% predicted within one year of follow-up), physiological signs of disease progression and radiological evidence of disease progression in patients with interstitial lung disease, occurring within the past year with no alternative explanation [1].

To date, there are no specific laboratory tests to diagnose both IPF and PPF. Early detection biomarkers and disease mechanisms are lacking.

For this reason, in order to investigate some characteristics of the disease and better understand the pathological steps leading to the onset of IPF and PPF, we evaluated two exosomal microRNA, respectively miR-21 and miR-92a, as possible diagnostic, prognostic and predictive biomarkers of the therapeutic’s response. Indeed, in the complexity of pulmonary fibrosis, the investigation of microRNA as possible biomarkers seems to be promising [2]. Actually, no enough studies provide reliable data on the function of the microRNA contained within exosomes [3].

Exosomes are small cell-derived vesicles present in various biological fluids [4]. They can carry multiple constituents of the naïve cells such as proteins, lipids, mRNA and microRNA and they could be released by different cell types. They could be found in most bodily fluids including blood, urine, saliva, amniotic fluid, and/or breast milk for example [5]. Initially it is believed to be a vehicle of cellular waste elements, even so exosomes play an important role in intercellular communication and be capable of influencing both physiological and pathological processes. Many molecular constituents of exosomes are associated with several diseases suggesting a role in a diagnostic tool.

Recently, microRNAs (miRNAs), a growing family of small non-coding RNAs, have gained significant attention for their work as post-transcriptional regulators of gene expression and control various cellular processes such as differentiation, proliferation, and cell–cell interaction [6]. To date, it is known that miRNAs dysregulations are linked to a wide spectrum of diseases [7–9] including diabetes [10], cancer [11], cardiovascular diseases [12] and fibrosing processes affecting various organs and tissues [13, 14].

Scientific research has recently focused on the link between miRNA dysregulation and the onset of disorders in fibrogenesis, or its progression, finding multiple findings in various pathologies such as IPF. The miRNA involved in IPF can easily be divided into profibrotic and antifibrotic, whose expression is increased (up-regulated) and decreased (down-regulated), respectively. Following these considerations, in our study we decided to evaluate the expression in the BAL of two exosomal miRNA (exo-miRNA), miR-21 identified as a pro-fibrotic miRNA [13, 15] and miR-92a considered to be an anti-fibrotic miRNA [16–18], with the aim to highlight any expression differences between IPF, progressive pulmonary fibrosis and not-progressive pulmonary fibrosis.

## Materials and methods

### Population

Thirty-three patients with idiopathic pulmonary fibrosis (IPF) and 33 patients with not-IPF pulmonary fibrosis were enlisted from the Institute of Respiratory Diseases, “Policlinico Riuniti” of Foggia, Italy.

Written informed consent was obtained from all subjects and an ethical approval was obtained from Ethics Committee of “Policlinico of Foggia” (n. 26/CE/2023). Protocols conformed to the principles of the Declaration of Helsinki.

The diagnosis of IPF was fulfilled according to the ATS/ERS/JRS/ALT 2022 guidelines [1].

The patients affected by not-IPF pulmonary fibrosis were divided as follows: 4 individuals affected by sarcoidosis, 10 patients by hypersensitivity pneumonitis (HP), 7 affected by not specific interstitial pneumonia (NSIP), 4 with pulmonary fibrosis in the context of rheumatological diseases, 1 subject with combined pulmonary fibrosis and emphysema (CPFE), 1 iatrogenic pulmonary fibrosis and 6 inpatients affected by interstitial lung disease not otherwise specified.

Regarding the initial diagnosis, individuals belonging to the not-IPF group were then divided according to disease progression into two groups: progressive pulmonary fibrosis (PPF) and not-progressive pulmonary fibrosis (nPPF).

This subdivision was carried out in accordance with the criteria recently proposed by Hambly et al. to define the progression of the disease [19]. All enrolled patients were naïve upon recruitment. 38 were non-smoker at the time of sample collection.

The population were therefore divided into three separate groups: i) IPF; ii) progressive pulmonary fibrosis (PPF); iii) not-progressive pulmonary fibrosis (nPPF).

All enrolled subjects underwent pulmonary function tests (PFT), walking test and bronchoscopy with bronchoalveolar lavage (BAL) sampling, after obtaining written consent to the procedure.

### Pulmonary Function Tests (PFT)

The respiratory function tests were carried out with a VMAX22 ENCORE spirometer and V62J plethysmographic cabinet, VYAIRE-USA, VMAX Carefusion software. Reporting data were accomplished in accordance with the ATS/ERS 1993 theoretical values reference document [20].

Global spirometry (with plethysmographic technique), through the measurement of static and dynamic volumes of the lung, allowed the diagnosis of the ventilatory deficit. The parameters analyzed were: percent forced vital capacity (FVC%) which represents the most widely used index to monitor the progression of IPF [1] and total lung capacity (TLC). To complete this examination, a diffusion lung carbon monoxide (DLCO) test was carried out to study the transfer of respiratory gases.

### Walking test

All patients underwent walking tests. The test was performed in a straight corridor with a rigid walking surface and a length of 30 m. Each patient was invited to walk for 6 min in self-pace mode and they decided the effort’s intensity as well as to slow down or stop along the way.

During the test the following criteria were monitored: heart rate, blood pressure, peripheral oxyhemoglobin saturation, dyspnea according to the Borgs’ scale and meters covered.

The walking test shows the submaximal patient’s effort, exercise capacity [21] and the meters covered that represent a useful parameter for assessing disease progression [1].

### Bronchoalveolar Lavage (BAL)

All patients underwent bronchoscopy. During the procedure BAL was collected. Partially the washing liquid was used to study lung cellularity; the remaining was aliquoted and stored at -80 °C for subsequent analyses.

### Exosome purification from BAL

Exosomes were isolated from BAL samples by ultracentrifugation [22]. BAL samples were diluted with PBS, centrifuged for 30 min at 2000 × g and then for 45 min at 12.000 × g at 4 °C. The obtained supernatants were filtered through a 0.22 µm filter and ultracentrifuged for two cycles of 70 min at 110.000 × g in order to concentrate the exosomes in the pellet. Precipitated exosomes were suspended in 200 µL of PBS and stored at -80 °C until use.

### Western blotting

The protein concentration of the exosomes was determined by Bradford analysis, as recommended by the manufacturer (BIORAD). 80 µg of proteins were subjected to electrophoretic migration in a 12% SDS-poly-acrylamide gel and transferred into PVDF membrane (BIORAD). The membrane was blocked with TBS/5% milk (Tris-buffered saline/0.1% Tween-20/5% low fat milk) and then incubated with the appropriate primary antibody diluted into blocking solution (CD81 (#10630D), Invitrogen; CD9 (#SC13118, Santa Cruz Biotechnology). After washings with TBS/0.1% Tween-20, the membrane was incubated for 1 h with the secondary antibodies, and it was diluted in the same buffer as previously described. Bands were visualized using ECL (BIORAD).

### Multiplex surface marker analysis

MACSPlex analysis was performed using MACSPlex Exosome Kit – human (Miltenyi Biotec, Bergisch-Gladbach, Germany), capable of detecting 37 surface exosomal epitopes and two isotypic controls.

Samples containing exosomes were processed as follows: exosomes (4–20 μg of protein) were diluted with MACSPlex buffer (MPB) to a final volume of 120 μl and 15 μl of MACSPlex exosome capture beads were added. After an overnight incubation in the dark, at room temperature (RT), on an orbital shaker (450 rpm), 500 μl of MACSPlex Buffer were added to each tube; the tubes were then centrifuged at RT at 3.000 × g for 5 min. The supernatant was aspirated and 5 μl of MACSPlex exosome detection reagent CD9, CD63, and CD81 were added to each tube. The tubes were incubated, away from light sources, for 1 h at RT in an orbital shaker (450 rpm). Subsequently, 500 μl of MACSPlex Buffer was added to each tube and incubated in the dark for 15 min at RT in an orbital shaker (450 rpm). Samples containing exosomes were centrifuged and the supernatant was aspirated, leaving about 150 μl in the tube. Flow cytometric analysis was performed at Facs Canto II followed by Kaluza Analysis 2.1 (Beckman and Coulter Life Sciences, CA, USA).

The concentration of the epitopes present on the exosome surface were obtained from the ratio between [beads + exosomes + Ab] / [beads + Ab] of the corresponding controls.

### RNA extraction

Total RNA was extracted from exosomes using TRIzol reagent (Thermo Fisher Scientific, Waltham, MA, USA), according to the manufacturer's protocol. RNA concentration and quality were measured using the NanoDrop 1000 spectrophotometer (Thermo Fisher Scientific). RNA purity was assessed by the absorbance ratio at OD_260_/OD_280_.

### Reverse transcription and miRNA expression analysis by qRT-PCR

Exosomal RNA was reverse transcribed using TaqMan MicroRNA RT kit (Thermo Fisher Scientific), according to the manufacturer's instructions. The identified miRNAs were evaluated by quantitative real time PCR (qRT-PCR) performed with TaqMan miRNA assay (Thermo Fisher Scientific) according to the manufacturer's instructions.

RNU6B was used as an endogenous control [23]. The reaction was carried out on the ABI-PRISM 7300 (PE Applied Biosystems). The relative quantification was carried out using the comparative method of 2^−ΔΔCt^ [24].

### Statistical analysis

Comparisons between groups were performed by ANOVA or Kruskal–Wallis test depending on whether the data were normally distributed or not. The data are presented as mean ± standard deviations (SD) or median ± range, depending on the normality of values.

Spearman's correlation was used to assess the correlations between relative miRNA expression and clinical and biological parameters. A *p*-value < 0.05 has been considered statistically significant. GraphPad Software (version 9.0, GraphPad Software) was used for the analysis.

## Results

### Population

The demographic and clinical data of the population studied are reported in Table 1.

**Table 1:** Clinical data of patients

	**TOTAL** (*n* = 66)	**IPF** (*n* = 33)	**PPF** (*n* = 19)	**nPPF** (*n* = 14)	***P*-value**	**Tukey ost-hoc**
Sex, % male	65	85	48	43	0.002**	IPF > PPF = nPPF
Age, years	66.0 ± 8.3	67.8 ± 7.1	65.5 ± 7.5	62.8 ± 11.1	0.170	-
Smoke, % smokers	42	56	26	29	0.059	-
*Functional respiratory data*						
FVC, %	74.6 ± 20.3	69.9 ± 16.4	87.2 ± 16.0	70.1 ± 27.2	0.01*	IPF > PPF > nPPF
DLCO, %	58.3 ± 19.4	53.9 ± 16.1	73.1 ± 23.0	52.0 ± 15.0	0.004**	IPF > PPF > nPPF
6MWT, meters	356.8 ± 137.5	376.0 ± 128.0	363.8 ± 166.7	264.6 ± 142.1	0.202	-

Summary of the main clinical data observed in patients

Data are reported as mean ± SD and were analyzed using ANOVA test. *p < 0.05 and **p < 0.01

*FVC* Forced vital capacity, *DLCO* Diffusing capacity for carbon monoxide, *6MWT* 6-min walking test

No significant differences were found for age, smoking’s history and meters covered in the walking test. Significant differences were found for the clinical values FVC%, DLCO% and for the demographic parameter gender.

### Exosomes characterization

**Fig. 1:**
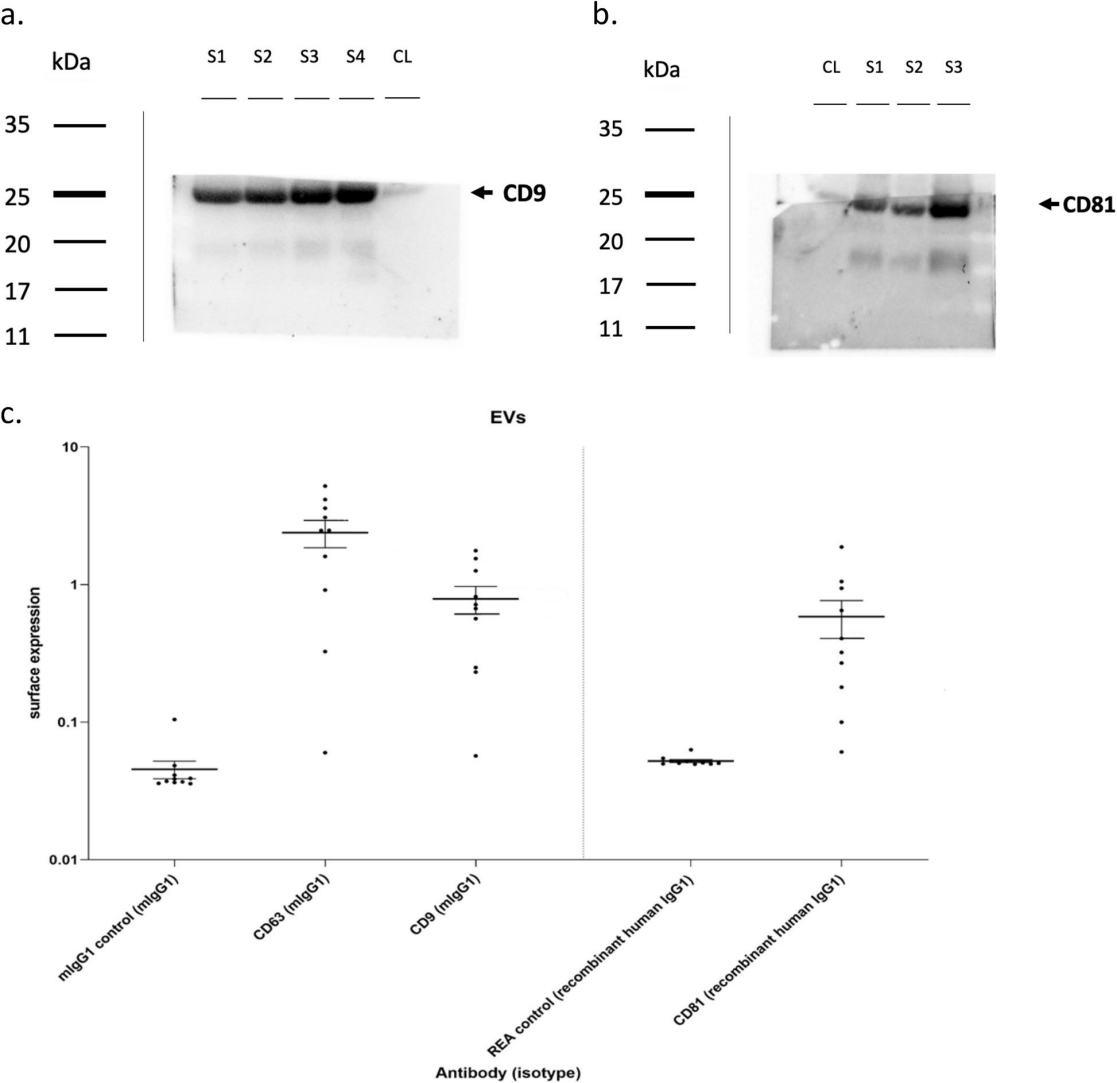
Exosomes characterization using western blot and flow cytometric analysis Characterization of isolated exosomes by Western Blotting analysis using primary antibodies directed to CD9, molecular weight 24 kDa (**a**) and CD81, molecular weight 22–26 kDa (**b**). Bands were obtained using an exposition of 25 s. Characterization of isolated exosomes by flow cytometric analysis (**c**) using mlgG1 and REA as isotypic controls. Blots were cut prior to hybridisation with antibodies during blotting. Full-length blots are presented in Supplementary Figure S1 S: samples; CL: cellular lysate

The characterization of the isolated exosomes was carried out by Western Blot analysis, in which 3 or 4 random samples were tested to evaluate the presence of CD9 and CD81 (proteins characteristic of the exosomal surface). The analysis clearly showed the presence of both CD9 and CD81 in all the analyzed samples (Fig. 1a and b). Blots were cut prior to hybridisation with antibodies during blotting. Full-length blots are presented in Supplementary Figure S1. Subsequently, a qualitative flow cytometric analysis was performed, Fig. 1c, that showing the presence of exosomal surface epitopes (CD9, CD69, CD81) on almost all the exosomes isolated from BAL compared to controls. Additional images of the Western Blot are included as Supplementary.

### Exo-miRNAs expression

All groups expressed profibrotic miR-21 demonstrated the nature of the underlying pathologies.

**Fig. 2:**
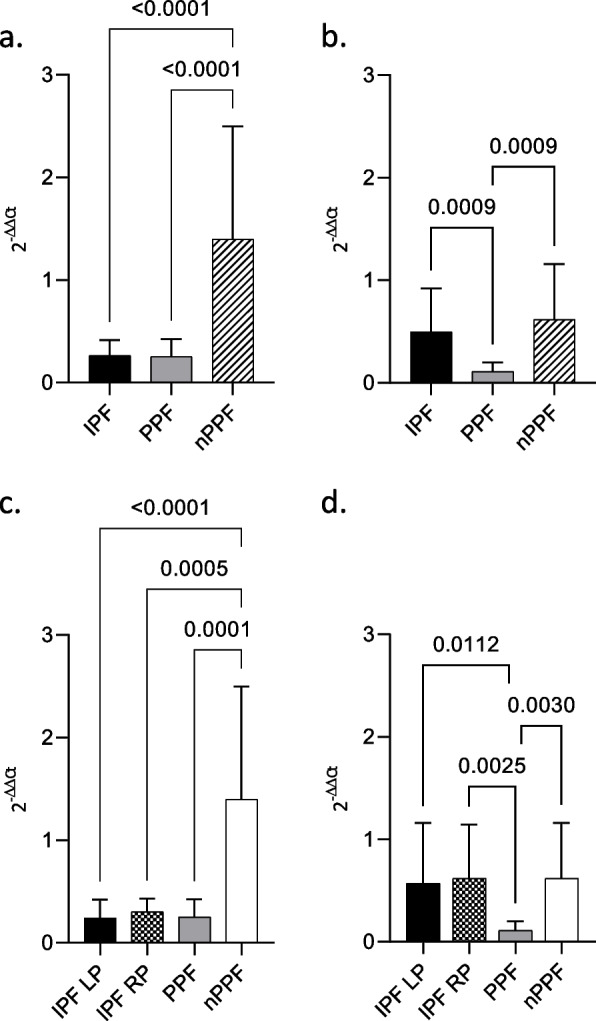
Exosomes-derived miRNAs expression qRT-PCR analysis of differentially expressed miRNAs in BAL exosomes from IPF, PPF and nPPF RNU6B was used as internal control. **a** miR-21; **b** miR-92a; **c** miR-21 (IPF group was split into RP and LP); d. miR-92a (IPF group was split into RP and LP). MiRNA expression is shown as fold change (2-ΔΔCt). *p*-value is shown only if there is a significant expression difference

Statistically significant differences in miR-21 expression were found between IPF and nPPF (*p* < 0.0001), and between PPF compared to nPPF (*p* < 0.0001) (Fig. 2a).

No statistically significant differences were found between IPF and PPF.

On the other hand, miR-92a showed a significant difference in expression between IPF and PPF (*p* = 0.0009) and between PPF and nPPF (*p* = 0.0009). There was no difference between IPF and nPPF (Fig. 2b).

In particular, miR-92a, known as an antifibrotic, according to our results, appears to be downregulated in progressive forms, both in comparison to IPF and to non-progressive ones. These results suggested that the protective effect of miR-92a action is less in progressive pulmonary fibrosis than in IPF or nPPF. This data underlines the protective role of miR-92a, which is more expressed in IPF or nPPF groups.

Furthermore, we decided to further divide the IPF group into two subgroups consisting of the “rapidly progressive” (RP) vs “slowly progressive” (LP) phenotypes. For both miR-21 subgroups, RP and LP, showed the same difference in expression in comparison to nPPF, already highlighted before the subdivision (Fig. 2c).

For miR-92a, either RP and LP showed a statistically significant difference compared to the PPF group, a difference already found before the subdivision into subgroups (Fig. 2d). There was no difference between LP and RP, neither in the miR-21 and miR-92a.

According to the reported data, miR-92a appeared to be more downregulated in progressive than not-progressive forms. This confirms that the difference in antifibrotic miRNA expression could be linked to the progression of the disease.

### Exo-miRNAs expression according to gender

**Fig. 3:**
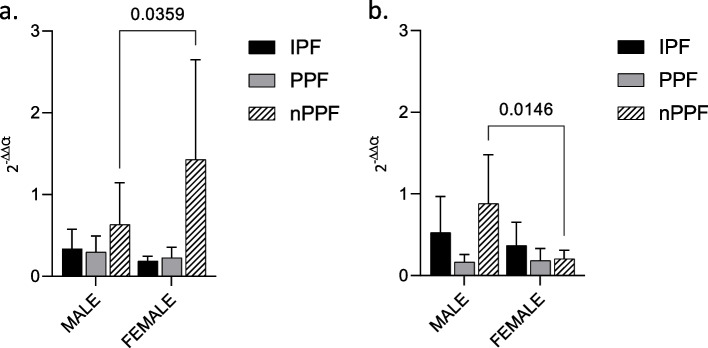
Exo-miRNA expression according to gender miRNA expression in BAL exosomes from IPF, PPF and nPPF divided according to gender (male or female). **a** miR-21; **b** miR-92a. RNU6B was used as internal control. MiRNA expression is shown as fold change (2-ΔΔCt). *p*-value is shown only if there is a significant expression difference

For both miRNAs studied, miR-21 and miR-92a respectively, a difference in expression according to gender (male or female) was found only in the nPPF group (Fig. 3).

No statistically significant difference in miRNAs expression was found among male and female in IPF or PPF population (Fig. 3).

In relation to that, both IPF and non-IPF progressive pulmonary fibrosis, gender does not appear to be greatly involved in determing.

the expression of profibrotic or antifibrotic factors. Nevertheless, in the nPPF group, gender could have an impact on disease progression. In particular, miR-21 expression was higher in females than in males (*p* = 0.0359) (Fig. 3a). Conversely, miR-92a was highly expressed in males than females (*p* = 0.0146) (Fig. 3b).

### Exosomal miRNAs expression according to age (threshold: 66 years, median at diagnosis)

**Fig. 4:**
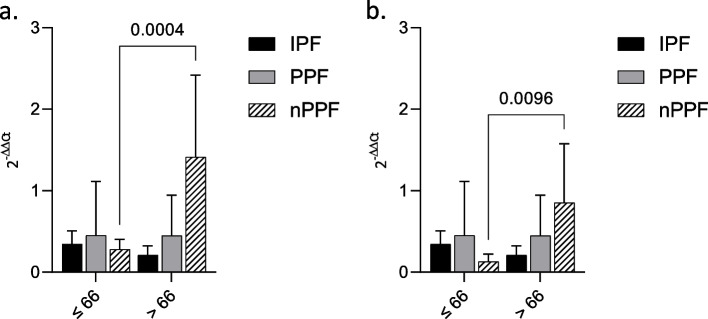
Exo-miRNA expression according to age. miRNA expression in BAL exosomes from IPF, PPF and nPPF divided according to age (threshold: 66). RNU6B was used as internal control. **a** miR-21; **b** miR-92a. MiRNA expression is shown as fold change (2-ΔΔCt). *p*-value is shown only if there is a significant expression difference

Both miR-21 and miR-92a a significant difference was found in the nPPF group based on age (threshold: 66 years). Most of all, miR-21 in the nPPF group was more expressed in subjects older than 66 years of age (*p* = 0.0004). The same holds for miR-92a, which was more highly expressed in older subjects in the nPPF group (*p* = 0.0096) (Fig. 4).

No statistically significant difference was found in IPF or PPF either for miR-21 or for miR-92a, therefore their expression was not affected by age.

### Exo-miRNA expression according to smoking history

**Fig. 5:**
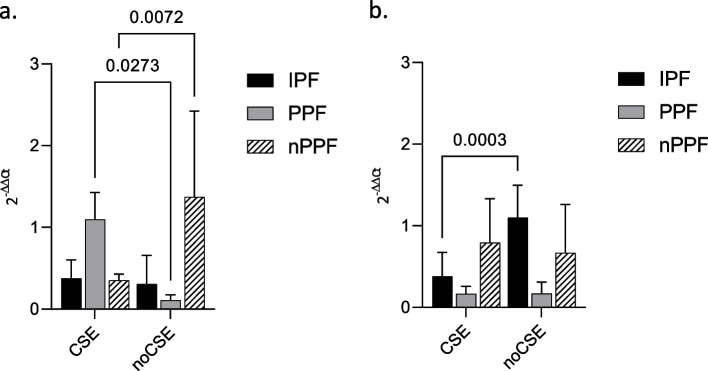
Exo-miRNA expression according to smoking history. miRNA expression in BAL exosomes from IPF, PPF and nPPF divided according to age (threshold: 66). RNU6B was used as internal control. **a** miR-21; **b** miR-92a. MiRNA expression is shown as fold change (2-ΔΔCt). *p*-value is shown only if there is a significant expression difference. CSE: cigarette smoke exposition

Finally, several statistically significant differences were found based on the smoking history. Especially for miR-21 which was markedly less expressed in the PPF group in non-smoking patients (*p* = 0.0273) and conversely, significantly less in the nPPF group in smoking patients (*p* = 0.0072) (Fig. 5a). On the other hand, miR-92a, appeared more expressed in the IPF group in non-smoking patients (*p* = 0.0003) (Fig. 5b).

### Clinical correlations

Correlation between the level of miR-21 and miR-92a and main functional respiratory data (FVC%, DLCO%, meters covered at 6MWT) are stated in Table 2.

**Table 2:** Correlation with the main functional respiratory data

	*DLCO, %*	*6MWT, meters*	*FVC, %*
**IPF**			
*miR-21*	0.478**	0.109	0.127
*miR-92*	0.043	-0.219	0.015
**PPF**			
*miR-21*	-0.030	0.199	-0.401*
*miR-92*	0.444*	0.547	-0.335
**nPPF**			
*miR-21*	-0.211	0.487	-0.546*
*miR-92*	0.187	-0.084	-0.012
**ALL GROUPS**			
*miR-21*	0.259*	0.194	-0.057
*miR-92*	0.197*	-0.096	0.045

*p*-value < 0.05 is marked in bold. *p ≤ 0.05; **p ≤ 0.001

*DLCO* Diffusing capacity for carbon monoxide, *6MWT* 6-min walking test, *FVC* Forced vital capacity

## Discussion

The term interstitial lung disease (ILD) encompasses a group of diffuse parenchymal lung diseases with varied clinical, radiographic, and pathologic manifestations reflecting their diverse underlying pathobiology.

Idiopathic pulmonary fibrosis is an interstitial lung disease with still unknown aetiology, which leads to rapid death within 2–3 years after diagnosis. IPF have a progressive fibrosing phenotype and is characterized by poor prognosis and quality of life.

However, other ILDs can also develop a progressive fibrosing phenotype and are therefore called progressive pulmonary fibrosis (PPF).

The complex pathogenesis of both IPF and PPF is not yet fully clarified. In this regards, exosomal microRNAs seem promising biomarkers [25].

Exosomes have emerged as essential actor impacting the progression of fibrotic diseases by transferring antifibrotic or profibrotic miRNAs to target cells and affecting pathological fibrogenesis.

The goal of our study was to elucidate the impact of pulmonary fibrosis related exosomal miRNA on fibrogenesis and progression. So, we evaluated the expression profile of two exo-miRNAs respectively, miR-21 with profibrotic function and miR-92a with antifibrotic function, on BAL within IPF, PPF and nPPF patients; in order to point out any differences in expression among groups.

It is known that miR-21 is a regulatory molecule in several physiological and pathological processes [26]. Recently, its role in fibrosing processes, including idiopathic pulmonary fibrosis, has appeared in studies conducted, both, on lung tissue from patients with IPF and in well-standardized models of murine pulmonary fibrosis. These studies have shown that, under the above circumstances, miR-21 is overexpressed and upregulated [13, 15].

In esteem, to context of fibrosing processes, including IPF, the role of miR-21 dysregulation as prognostic factors has been postulated [27].

In our study the analysis of miR-21 expression showed significant differences between nPPF compared either to IPF and PPF, demonstrating that its expression could be determined in the differential diagnosis between progressive and non-progressive pulmonary fibrosis.

Therefore miR-21, based on our data, seems to be useful as diagnostic biomarker of IPF. It could be used as predictive biomarker of progression in our population, as it shows significant differences between progressive and non-progressive fibrosis.

Nonethless, the dysregulation of miR-92a and its role in pulmonary fibrosis [17] has been extensively explained by Berschneider et al. The authors for the first time demonstrated the inverse correlation between the expression of miR-92a and WISP1 protein in idiopathic pulmonary fibrosis. In particular, they found that, in IPF, miR-92a is downregulated, while the expression of the WISP1 protein is increased [18]. WISP1 has been demonstrated to contribute to IPF pathogenesis and this gene is found to contain target sites for miR-92a in the 3’-UTR region. Based on it, the regulatory role of miR-92a on WISP1 expression in reversing fibrotic phenotype emerges.

Thereby the anti-fibrotic role of miR-92a turn out alongside the role of miRNAs as potential therapeutic targets in pulmonary fibrosis.

Our study highlighted a statistically significant difference between miR-92a expression between IPF and PPF. In particular, miR-92a appears to be more downregulated in PPF than IPF. Its protective role was less significant in progressive forms compared to IPF, defining the paradigm of chronic pulmonary fibrosing diseases. This result is important because underlines the aggressive biological behavior of progressive pulmonary fibrosis, regardless of their initial diagnosis.

A statistically significant difference in miR-92a expression was also found between progressive pulmonary fibrosis and the group of not-progressive pulmonary fibrosis, in which miR-92a is expressed to a greater extent. The result underlines the protective role of miR-92a, which is widely expressed and in a statistically significant way, in the group of pulmonary fibroses that do not progress. Therefore the potential of miR-92a become relevant as a predictive factor of progression to the diagnosis of pulmonary fibrosis emerges.

Concerning the statistically significant gender difference within our population, the study of the expression of the two mRNAs was conducted on the whole population in order to reveal the possible role of gender in their expression.

The analysis of our data shows that gender affects the expression of both miRNAs studied, but only within the nPPF group. No gender differences were found in either the IPF or PPF groups.

It is important to clarify this data because there is a male gender prevalence in IPF [27]. Neverthless, our data demonstrated a higher expression of miR-21, profibrotic, in female of the nPPF group. On the other hand, miR-92a, with a protective effect, seems to be more expressed in men of the nPPF group.

As a consequence, the pathogenesis and/or progression of fibrosis could be attributable to a different expression of miRNAs due to gender.

An analysis of miR-21 and miR-92a expression according to gender was also performed in the IPF group. A not-statistically significant trend was found in miR-21 expression between males and females; however, trend does not appear to be attributable to gender, furthermore the number of female subjects affected by IPF is seems to be small. If this data were confirmed by further studies, factors that promote the expression of miR-21 would have to be searched within the male population affected by IPF, currently only presumed, since the etiology of the disease is unknown compared to females. For example, these elements could be linked to risk factors for pathology, to which male individuals are more exposed, for instance cigarette smoking, occupational exposure to dust and metals [28]. Currently in both IPF and not-IPF pulmonary fibrosis, regardless of their progression, gender is not involved in influencing the expression of profibrotic or antifibrotic factors [29, 30].

Additionally, to verify the role of age in the differential expression of microRNAs, we conducted the analysis of their expression by dividing the population into two subgroups based on age by using the median at diagnosis (66 years) as a threshold.

For both miRNAs analysed, the only statistically significant differences found were within the nPPF group.

Our data suggest that age does not influence the expression of miR-21 and miR-92a in IPF or non-IPF progressive pulmonary fibrosis. Therefore, it does not bias for one or the other pro- and antifibrotic element. Regarding IPF, considered a disease of old age [31], we could state that it is not attributable to an age-related increase in the expression of the profibrotic miR-21, or to differences in the expression of the antifibrotic miR-92a. The role of age could probably be attributed to other elements, such as the alteration of the regenerative capacities of the alveolar epithelium due to aging.

In our study we emphasize the reciprocal trend of miR-21/miR-92a expression, discovering a possible imbalance towards the profibrotic activity of one versus the antifibrotic activity of the other ones.

The investigation was carried out in all analyzed group highlighting a direct correlation. Since these are miRNAs with opposite biological function in the pathogenesis of the disease, an inverse correlation would be hypothesized between them [13, 15, 17]. Instead, our results show a concordant trend of the two microRNA in all group. This finding could suggest that the increase of one of the two miRNAs triggers an endogenous counter-regulation mechanism, that attempts to balance the two conditions, or there could be a different stimulus to unbalance towards one, or the other event. Furthermore, their direct correlation, against an opposite function, suggests the miRNAs are probably not the only factors determining the pathogenesis but more complex processes could interfere with them.

The clinical correlations were also studied between the expression of the two miRNAs and the clinical parameters at diagnosis.

The analysis reveals no statistically significant trend between the increase in miR-21 expression and the decline in FVC% in the progressive fibrosis. As a results, for those individual undergoing disease progression, elevated miR-21 expression at diagnosis time could correlate with a greater than predicted decline in FVC%, and with a worsening lung’s function.

In this type of progressive fibrosis, the enhanced activity of miR-21 at diagnosis could correlate and be revealed with an early deterioration of lung function. If validated by future studies, this data, would be very important, because the dosage of miR-21 at the time of diagnosis could provide a data with a prognostic value predictive of progression in not-IPF fibrosis; these would allow early selection of patients who need to undergo more check-ups tightened or more intensive treatments.

Finally, the study showed that, likeness other biological fluids, BAL contains exosomes which contains miRNAs as cargo [3, 32, 33].

Based on the data reported, BAL does not currently appear to be useful in the differential diagnosis between IPF and not-IPF pulmonary fibrosis, since it would not be a determinant in equivocal cases. Therefore, in doubtful cases, research for these biomarkers in the BAL does not allow us to avoid surgical biopsy.

The role of BAL, currently limited to diagnostic purposes, could be useful in the minimally invasive search for further identify locally expressed progression markers in those individuals that could undergo intensive treatment.

New studies on free microRNA in BAL will be needed in the future to further clarify the diagnostic and prognostic role of this minimally invasive method.

Overall, this is just a first study that compare two BAL exosomal miRNAs from IPF, PPF and nPPF to suggest the unique miRNAs signature that could be used as potential biomarker to identify pulmonary fibrosis progression and could be developed as therapeutic targets. Further studies are needed to validate our findings in larger cohorts and to understand the role of exosomal miRNAs in pulmonary fibrosis development, progression and severity.

### Limitations of the study

Due to absence of specific laboratory tests able to diagnose both IPF and PPF, our clinical study relies on relatively small population and matching was not perfect. However, we evidenced statistically significant differences in miRNA expression, suggesting that the variations are strong enough to be detected in this small cohort.

For this study, the miRNAs analysed derive from a careful study of the literature and were selected after numerous researches about the topic. Certainly, further studies, through NGS analysis, will be required to confirm the highlighted.

## Conclusions

Thanks to the obtained results, we could state that the search for specific diagnostic biomarkers of IPF in BAL seems to be promised.

The need to identify markers of progression derives from the considerable variety of the clinical course of patients with IPF who may undergo a gradual and slow progression, or a very rapid decline, with early mortality as well as periods of relative clinical stability. It is very difficult to identify patients at high risk of progression at the time of diagnosis, therefore it is necessary to study predictive models of risk based on clinical or biochemical parameters, such as miRNA.

In our study, the research for progression biomarkers on BAL highlighted the role of miR-92a as potential progression biomarker. Indeed, the downregulation of miR-92a expression seems to be related to the progression of fibrosis, regardless of the initial diagnosis; on the other hand, the increase in its expression exerts a protective effect and characterizes the forms of pulmonary fibrosis that hardly progress. The expression of miR-92a allows the early identification of patients with progressive disease, who are candidates for antifibrotic therapy from the beginning.

Moreover, according to our results, the expression of exosomal miR-21 in the BAL could be decisive in the differential diagnosis between progressive pulmonary fibrosis (IPF and non-IPF) and non-progressive pulmonary fibrosis.

To date, according to our knowledge, there are no similar studies in literature showing the use of these two microRNAs as diagnostic biomarkers in progressive and non-progressive pulmonary fibrosis. There are various studies showing dysregulated miRNAs in lungs of patients with idiopathic pulmonary fibrosis, although just few of them are indicative of the profound role of miRNAs in the progression of pulmonary fibrosis [34].

### Supplementary Information

**Additional file 1.**

## Data Availability

The data presented in this study are available on request from the corresponding author.

## Abbreviations

6MWT: 6-Minute Walking Test
BAL: Bronchoalveolar Lavage
CPFE: Combined Pulmonary Fibrosis and Emphysema (CPFE)
DLCO: Diffusing Capacity for Carbon Monoxide
ECM: Extracellular Matrix
EMT: Epithelial Mesenchymal Transition
exo-miRNA: Exosomal microRNA
FVC: Forced Vital Capacity
HP: Hypersensitivity Pneumonitis (HP)
IPF: Idiopathic Pulmonary Fibrosis
LP: Slowly progression
miRNA: MicroRNA
nPPF: Not-Progressive Pulmonary Fibrosis
NSIP: Not Specific Interstitial Pneumonia
PPF: Progressive Pulmonary Fibrosis
qRT-PCR: Quantitative Real Time PCR
RP: Rapidly progression
UIP: Usual Interstitial Pneumonia
