# Supplementary material for: Searching for airways biomarkers useful to identify progressive pulmonary fibrosis
Source: BMC Pulm Med. 2023 Oct 26;23:407. doi: 10.1186/s12890-023-02714-y (PMC10605223; doi:10.1186/s12890-023-02714-y)
Supplement: Supplementary file 1 — Additional file 1. [file 12890_2023_2714_MOESM1_ESM.docx]

**SUPPLEMENTARY**

**Figure S1.** Characterization of exosomes by Western Blotting

Original and unprocessed western blot image. CD9 and CD81 are labeled as in the main text.

ALIX and GAPDH are also highlighted, which have been executed simultaneously, but are not shown in the main text.

Bands were obtained using an exposition of 30s.

Blots were cut prior to hybridisation with antibodies during blotting. The entire image was achieved by reassembling, like a puzzle, the membranes that were cut before hybridization.

Specific antibodies were used to incubate each cut membrane. Different concentrations were used for all antibodies. After 25s of exposure, the membranes were first detected individually. Afterwards, they were put together, like a puzzle, and detected together after 30s of exposure. However, since antibodies are all different, the exposure time for each membrane was different. That's why there are some black spots on the right side of the figure in the entire image (CD9).

S: sample; CL: cellular lysate.

**Additional Western Blot images**

**A.** CD9_exposition 20s, **B.** CD9_exposition 25s, **C.** CD81_exposition 20s, **D.** CD81_exposition 25s, **E.** ALIX_exposition 25s,

**F.** ALIX_exposition 30s, **G.** GAPDH_exposition 25s, **H.** GAPDH_exposition 30s, **I.** ALL MEMBRANES TOGHETER_exposition 30s
